# The local and long‐range input landscape of inhibitory neurons in mouse auditory cortex

**DOI:** 10.1002/cne.25437

**Published:** 2022-12-01

**Authors:** Gen‐ichi Tasaka, Claudia Maggi, Elham Taha, Adi Mizrahi

**Affiliations:** ^1^ The Institute of Life Sciences The Hebrew University of Jerusalem Jerusalem Israel; ^2^ The Edmond and Lily Safra Center for Brain Sciences The Hebrew University of Jerusalem Jerusalem Israel

**Keywords:** auditory cortex, inhibitory neurons, monosynaptic rabies tracing

## Abstract

Roughly 20% of the neurons in the mouse cortex are inhibitory interneurons (INs). Of these, the three major subtypes are parvalbumin (PV), somatostatin (SST), and vasoactive intestinal polypeptide (VIP) expressing neurons. We used monosynaptic rabies tracing to compare the presynaptic input landscape onto these three IN subtypes in the mouse primary auditory cortex (A1). We compared both local patterns of monosynaptic inputs as well as long‐range input patterns. The local monosynaptic input landscape to SST neurons was more widespread as compared to PV and VIP neurons. The brain‐wide input landscape was rich and heterogeneous with >40 brain regions connecting to all the three INs subtypes from both hemispheres. The general pattern of the long‐range input landscape was similar among the groups of INs. Nevertheless, a few differences could be identified. At low resolution, the proportion of local versus long‐range inputs was smaller for PV neurons. At mesoscale resolution, we found fewer inputs from temporal association area to VIP INs, and more inputs to SST neurons from basal forebrain and lateral amygdala. Our work can be used as a resource for a quantitative comparison of the location and level of inputs impinging onto discrete populations of neurons in mouse A1.

## Graphical abstract

We used monosynaptic rabies tracing to compare the presynaptic input landscape onto three inhibitory neuron subtypes, PV, SST, and VIP, in the mouse primary auditory cortex (A1). A leakless mutant of TVA receptor allowed us to compare both local patterns of monosynaptic inputs as well as long‐range input patterns. Our work can be used as a resource for a quantitative comparison of the location and level of inputs impinging onto discrete populations of neurons in mouse A1.

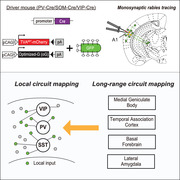

## 1 INTRODUCTION

Roughly 20% of the neurons in the mouse cortex are inhibitory interneurons (INs). Cortical INs have been the focus of intense research with an ever‐increasing amount of knowledge about their structure, connectivity, and function (Tremblay et al., 2016). The functions of cortical INs are many, ranging from the shaping incoming signals and modulating local computations (Isaacson & Scanziani, 2011), through having a key role in development and plasticity (Takesian et al., 2018). Abnormal development and function of interneurons are also central to numerous brain pathologies (Hattori et al., 2017; Marín, 2012). Thus, understanding cortical INs remains a central challenge in order to understand cortical function in the mammalian brain.

INs classification has revealed the presence of three nonoverlapping cell subtypes—parvalbumin (PV), somatostatin (SST), and vasoactive intestinal polypeptide (VIP) expressing neurons (PV, SST, and VIP, respectively) (Xu et al., 2010). Although these three nonoverlapping subtypes are present across the cerebral cortex, clear differences among brain areas exist (Tasic et al., 2018). For example, several studies provided evidence for differences in the connectivity and function of INs localized in different brain regions (Barbour & Callaway, 2008; Mesik et al., 2015). Thus, areal‐specific characterization is important in order to understand the specific function subserved by that area. However, the majority of research on INs in the rodent brain has been focused on the somatosensory and visual cortices (Tremblay et al., 2016). Nevertheless, the functional role of different IN subtypes in A1 is steadily growing (Cooke et al., 2020; Gothner et al., 2021; Liang et al., 2019; Moore & Wehr, 2013; Phillips & Hasenstaub, 2016; Schneider et al., 2014; Seybold et al., 2015; Studer & Barkat, 2022). Here, we focus on the connectivity of INs in mouse primary auditory cortex (A1).

It has been technically difficult to reveal the full connectome onto and from single neurons because these span multiple spatial scales. A full connectivity landscape should eventually be determined by identifying the synaptic inputs from upstream neurons and a neuron's synaptic outputs to postsynaptic neurons downstream. Delineating the complete connectivity matrix of any neuron would necessitate both local as well as long‐range information. Unfortunately, to date, there is no single method that can unveil such information for neurons in the mammalian neocortex. In turn, cortical connectivity data rely on information collected from a variety of methods, each with its own advantages and disadvantages. For example, the local connectivity of INs (both inputs and outputs) in the auditory cortex has been revealed using slice electrophysiology and classical anatomical tracing (Levy & Reyes, 2012; Takesian et al., 2018). Long‐range connectivity of axonal targets from neurons in auditory cortex has been revealed by classical tracing as well as other methods like RNA barcoding (Chen et al., 2019; Oh et al., 2014). These latter methods reveal the mesoscale connectome by large brush strokes, yet bear little information regarding synaptic specificity. The synaptic connectivity matrix of most areas in the mammalian brain, and particularly so the auditory cortex, remains an unfinished puzzle to complete.

Here, we used monosynaptic rabies virus tracing to study the local as well as the brain‐wide input landscape onto the three main subpopulations of INs in A1 of the mouse (i.e., PV, SST, and VIP). Recently, a similar study has been conducted in the barrel cortex, whereas several differences were detected within the brain‐wide input landscape of those INs (Wall et al., 2016). In A1, one study by Nelson and Mooney (2016) also traced IN subtypes using monosynaptic rabies virus tracing but focused mainly on inputs from the basal forebrain (BF), whilst the remaining brain‐wide data were studied only qualitatively or partially. Very recently, a rabies study traced the inputs from excitatory versus inhibitory neurons in A1, showing largely similar patterns of inputs to excitatory and inhibitory neurons (Zhao et al., 2022). Here, we increase the resolution one step further by discriminating among three different IN subpopulations, which have been suggested to play distinct roles in A1. We present a quantitative, brain‐wide comparative analysis of the brain regions sending monosynaptic inputs onto the three INs subtypes of mouse A1.

## 2 RESULTS

### 2.1 Monosynaptic rabies tracing of three inhibitory neuron subtypes in auditory cortex

**FIGURE 1:**
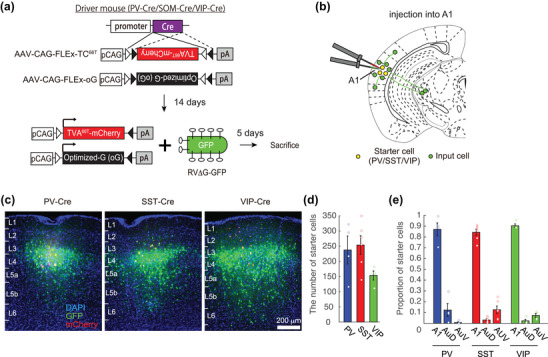
Rabies monosynaptic retrograde tracing from different types of inhibitory cells: (a) overview of rabies monosynaptic retrograde tracing components. TVA^66T^ was used for minimal leakage; (b) schematic of rabies tracing experiments. Yellow cells indicate the location of starter cells. Green cells indicate the monosynaptic inputs to the starter cells; (c) representative fluorescent micrographs of coronal brain slices containing injection sites in primary auditory cortex (A1). Left: parvalbumin (PV)‐Cre, middle: somatostatin (SST)‐Cre, right: vasoactive intestinal polypeptide (VIP)‐Cre. Scale bar, 200 µm; (d) quantification of the absolute number of starter cells. The total number of starter cells from PV‐Cre, SST‐Cre, and VIP‐Cre mice are not different (PV‐Cre, *N* = 4 mice; SST‐Cre, *N* = 6 mice; VIP‐Cre, *N* = 4 mice); (e) fraction of starter cells in subregions of the auditory cortex. The majority of starter cells are in A1. AuD, dorsal auditory cortex; AuV, ventral auditory cortex

We studied both the local and the brain‐wide monosynaptic input landscape onto three IN subtypes of mouse A1. We focused on the main IN subtypes—PV, SST, and VIP—which are thought to comprise >95% of all cortical inhibitory neurons (Rudy et al., 2011). To target these interneurons with high specificity, we used three transgenic knock‐in mouse strains that express Cre recombinase under the specific promoters of PV, SST, or VIP (Hippenmeyer et al., 2005; Taniguchi et al., 2011). We injected a mixture of two Cre‐dependent AAV helper viruses (AAV2‐CAG‐FLEx‐oG and AAV2‐CAG‐FLEx‐TC^66T^) into A1, thus enabling monosynaptic rabies tracing from these three populations of INs as starter cells. Figure 1 shows the 19‐day protocol and viruses used (Figure 1a), a schematic of injection site and labeling outcomes (Figure 1b), and representative fluorescent micrographs from the injection site (i.e., A1) of all three mouse strains (Figure 1c). Under these conditions, starter cells are either PV, SST, or VIP and express both mCherry and green fluorescent protein (GFP) (Figure 1b,c, yellow labeling). Cells expressing GFP but not mCherry are presynaptic neurons to the starter cell population.

We note that it is also possible that Cre‐positive cells will express mCherry and GFP (but not oG) and will be mistaken for starter cells or express oG and GFP (but not mCh) and would be “secondary starter cells”. This sort of noise in our system was likely minimal as assessed by (1) no labeling at all when viruses were injected to control mice that did not express Cre recombinase (Figure S1a); (2) ∼100% overlap between mCherry expressing neurons and interneuron cell type, which we assessed only in the PV‐Cre strain (Figure S1b; see also (Ma et al., 2021) for exhaustive controls); (3) neurons expressing mCherry but not GFP were rare and limited to the local injection site. These few cells are potential starter cells that were not infected by the rabies virus, and we cannot rule out their contribution to the results. Under these conditions, presynaptic neurons are found both locally (as in Figure 1c) and in distant brain regions across the brain (see below).

We injected a total of 14 mice and traced the brain‐wide monosynaptic input landscape from a total of 3163 starter cells. The number of starter cells in different mice varied considerably (range: 111–350 cells) but was not significantly different among the different strains (Figure 1d; PV‐Cre, *N* = 4 mice, *n* = 238 ± 92 starter cells; SST‐Cre, *N* = 6 mice, *n* = 254 ± 75 starter cells; VIP‐Cre, *N* = 4 mice, *n* = 154 ± 30 starter cells; Kruskal–Wallis test, *p* = .14). The total number of presynaptic neurons strongly depends on the number of starter cells. Indeed, in our data, the Pearson correlation between the “number of starter cells” and “total number of GFP^+^ cells” was strong (*R* = .71, *p* = .004), indicating that the rabies labeling method is reliably quantifiable using our data. This correlation was also true when considering local inputs alone (*R* = .76, *p* = .001), or long‐range inputs alone (*R* = .62; *p* = .018). In other words, brains with more starter cells will reliably have more GFP^+^ neurons. Critically, however, we found no correlation between the “number of starter cells” and the total convergence index (CI), which is defined as the number of total input neurons in a brain per starter cell (Pearson correlation between “number of starter cells” and “CI”, *R* = −.14, *p* = .6). This latter result is important for arguing that the number of starter cells per mouse did not bias the counts of neurons, and hence, the distribution of the presynaptic landscape in our data set.

Since our data depend on stereotactic viral injections, we cannot rule out small biases in spatial distributions of starter cells in any cortical axis due to the injection procedure or infectivity of the viruses (e.g., due to the differential tropism or spread). Nevertheless, in our data set, we only included mice in which the site of injection was in the center of A1, and the majority of starter cells were verified as being confined to the anatomical limits of A1 (Figure 1e; PV‐Cre, 87.0%; SST‐Cre, 84.3%; VIP‐Cre, 90.4%). We detected relatively few of the starter cells in the two immediate cortices adjacent to A1 (i.e., dorsal auditory cortex—AuD and ventral auditory cortex—AuV) (Figure 1e). At the end of the 19‐day protocol, we prepared the brains for histology, which were then sliced in full at 50 µm resolution in the coronal axis. All slices were then imaged serially using a fluorescent microscope, and all starter cells as well as GFP^+^ neurons were counted and registered to their exact anatomical location within the brain (see Section 4 for more details).

**FIGURE 2:**
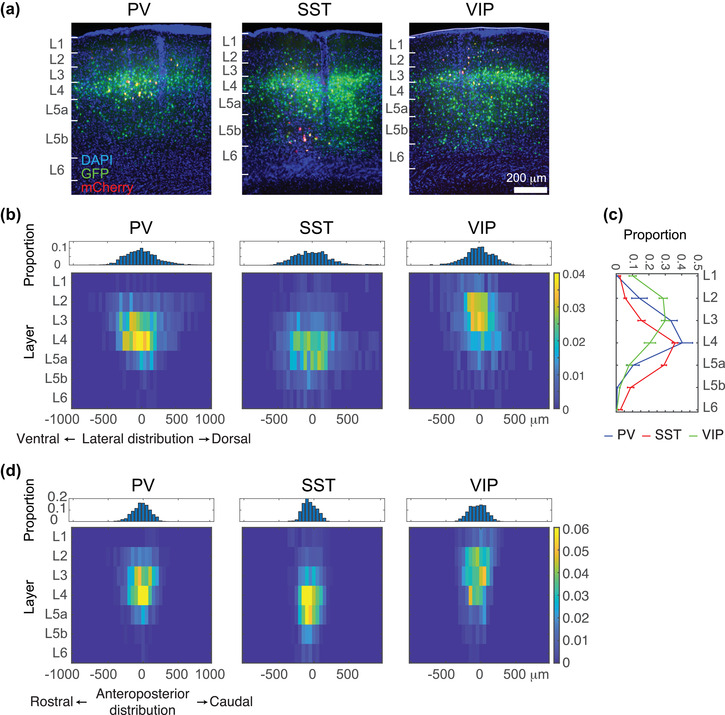
Spatial analyses of starter cells from parvalbumin (PV), somatostatin (SST), and vasoactive intestinal polypeptide (VIP) cells in A1: (a) representative fluorescent micrographs from the injection sites in A1. Scale bar, 200 µm; (b) top: the fraction of the lateral distribution of starter cells; each bin size is 50 µm; bottom: heat maps of the distribution of starter cells along the *D*–*V* axis; (c) the fraction of the layer distribution of starter cells. Error bars show *SEM*; (d) top: the fraction of the anteroposterior distribution of starter cells. Each bin size is 50 µm; bottom: heat maps of the distribution of starter cells along the rostral–caudal axis

To evaluate the distribution of starter cells within the injection site and along the cortical column axis (i.e., from pia to the white matter), we aligned the data by starter‐cell location, keeping their layer position and centering the whole population along the ventral–dorsal axis of the cortex (following methods by (DeNardo et al., 2015)). This alignment shows that PV and SST starter cells were distributed across all cortical layers with a peak around the middle layers (Figure 2a–c; PV and SST). The distribution of VIP starter cells peaked at more superficial cortical layers (Figure 2b,c; VIP). Analysis of the starter cell population along the anterior–posterior axis revealed similar distributions (Figure 2d). This distribution of starter cells is generally consistent with the known layer distributions of INs in A1 of adult mice (Ouellet & de Villers‐Sidani, 2014). In addition, the spatial distribution of starter cells, which is dominated by factors like injection volume, location, and diffusion, was largely homogenous with respect to the center of injection. More specifically, the number of starter cells dropped rapidly and symmetrically from its central peak to ∼0 within a radius of ∼0.5 mm (Figure 2b,d shows that no starter cells were found within ±1 mm of injection along any direction). Thus, our starter cell population was densest at the center of A1 yet sampled major portions of it.

### 2.2 Local input connectivity onto INs—SSTs receive spatially wider inputs

Traditionally, local synaptic connectivity is analyzed by methods like electrophysiology and photo‐stimulation (Luo et al., 2018). These methods have the power to reveal connectivity at high resolution, with reference to the dendritic morphology of individual neurons as well as the synaptic sign and strength (Levy & Reyes, 2012; Oviedo et al., 2010; Oviedo, 2017). While the advantages of rabies for tracing long‐range connectivity are rather established, its advantages for tracing local connectivity are less established, and far fewer studies have focused on local connectivity profiling using rabies (DeNardo et al., 2015). In fact, earlier work studying local connectivity using rabies tracing argued that the interpretation of rabies experiments of local connectivity should be done with caution because the reagents used at that time were shown to be locally leaky (Watabe‐Uchida et al., 2012). In this respect, the development of a mutant variant of the TVA receptor (TVA^66T^) proved to be far less leaky in the local circuit (Miyamichi et al., 2013). Another reason for taking a cautious approach with regard to the analysis of local connectivity analyses with rabies was the technical difficulty to accurately count single cells in densely labeled photomicrographs. This limitation is now alleviated by software that counts cells based on machine learning algorithms with high precision (Berg et al., 2019) (Figure S2). Thus, by (1) using reagents with nearly no leakiness, (2) post hoc evaluation of the exact locations of starter cells, and (3) combining manual and software‐based image analysis, we analyzed the local connectivity onto the INs of A1.

**FIGURE 3:**
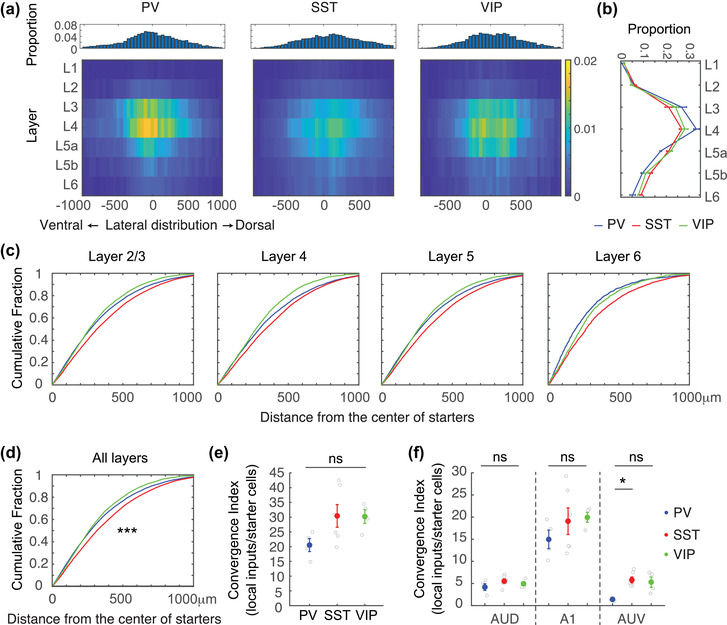
Spatial analyses of input cells from parvalbumin (PV), somatostatin (SST), and vasoactive intestinal polypeptide (VIP) cells in A1: (a) top: the fraction of the lateral distribution of input cells; bottom: heat maps of the distribution of input cells along the *D*–*V* axis; (b) the fraction of the layer distribution of input cells. Error bars show *SEM*; (c) the cumulative fraction of the pairwise distance of input cells from starter cells along the *D*–*V* axis in different layers; (d) the cumulative fraction of the pairwise distance of input cells from starter cells along the *D*–*V* axis in all layers. SST cells have a wider distribution of input cells than PV and VIP cells; (e) convergence index (CI) from all subregions of the auditory cortex; (f) CI from subregions of the auditory cortex

Local inputs to the three IN cell types were distributed in all cortical layers of A1 (Figure 3a,b). The distribution did not strictly adhere to the distribution of starter cells (i.e., compare parts (c) of Figure 2 to (b)). To evaluate the spatial distribution of inputs, we calculated the pairwise distance between all possible starter cells and GFP^+^ pairs. Within 1 mm across A1, the cumulative distance from input pairs of GFP^+^‐to‐PV and GFP^+^‐to‐VIP starter cells was similar. However, input to the SST starter cells was distinct. Specifically, the distribution of distances of GFP^+^‐to‐SST starter cell pairs was wider than that of the PV and VIP groups. This difference was evident in all layers individually (Figure 3c) and in A1 as a whole (Figure 3d, ks‐test; *p* < .0001 for all combinations). Similar results were obtained in the anterior–posterior axis (Figure S3a,b, ks‐test; *p* < .0001 for all combinations).

While the spatial distribution of local inputs was distinct in the SST group, the total number of input cells per starter cell, calculated as CI, was not significantly different across all three cell types in all brain regions (Figure 3e, Kruskal–Wallis test, *p* = .10) and A1 (Figure 3f, Kruskal–Wallis test, *p* = .33). There was a significant difference in AuV (Figure 3f, *p < .05, post hoc Tukey's HSD after significant Kruskal–Wallis test), which might be due to the smaller number of starter cells leaking to AuV in the PV group (Figure 1e). In summary, the main difference that we found in the local connectivity landscape of INs in A1 was a wider spread of the presynaptic landscape onto SST INs. This wider pattern of local connectivity may contribute to the functional role of SST INs in A1's local inhibition (Kato et al., 2017; Lakunina et al., 2020), and to their delayed responses as compared to other inhibitory cell types in A1 (Li et al., 2015).

### 2.3 Long‐range connectivity onto different interneurons is generally similar

**FIGURE 4:**
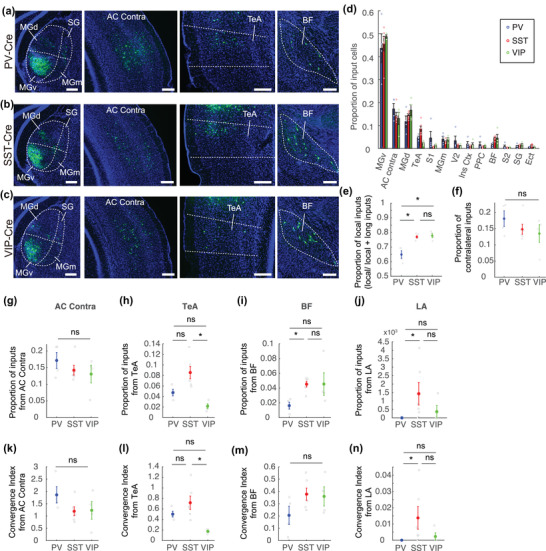
Long‐range input landscape of parvalbumin (PV), somatostatin (SST), and vasoactive intestinal polypeptide (VIP) cells in A1: (a–c) representative micrographs from select input regions into PV, SST, VIP cells in A1. Scale bar, 200 µm; (d) the fraction of long‐range input cells into each inhibitory cell type in A1. Only regions that received more than 0.2% of the total input fraction are shown. Local input cells (AuD, A1, and AuV) are excluded in this analysis; (e) the proportion of local input cells among all inputs, including long‐range inputs; although the majority of inputs are from the local vicinity in all three groups, SST and VIP neurons receive local inputs more dominantly than PV neurons; (f) the proportion of inputs from the contralateral hemisphere. (g–n) The proportion of inputs and the convergence index in the contralateral auditory cortex (g and k), the temporal association cortex (h and l), the basal forebrain (i and m), and the lateral amygdala (j and n). The convergence index is the number of input cells per starter cell

A main strength in using rabies tracing as compared to other methods is the ability to reveal the long‐range monosynaptic input landscape onto genetically accessible neurons, in vivo (Callaway & Luo, 2015). To quantify the long‐range landscape of inputs, we counted the number of all GFP^+^ neurons in each mouse across the whole brain. All brain regions with GFP^+^ neurons were manually identified by registering the images to the Paxinos and Franklin atlas of the mouse brain (Paxinos & Franklin, 2019). The number of cells in each region was scored by combining manual and semiautomatic counting (see Section 4). Figure 4a–c shows representative examples of micrographs taken from several brain regions with relatively high numbers of GFP^+^ cells labeled with rabies tracing.

We found that an average of 35 ± 7 long‐range brain areas were labeled with GFP^+^ cells in each animal. These data are largely consistent with our recent tracing results which were limited to excitatory neurons (Tasaka et al., 2020), and in support of recent data comparing excitatory and inhibitory neurons (Zhao et al., 2022). The total number of brain regions with labeled GFP^+^ cells per mouse was not statistically different among the experimental groups (one‐way ANOVA, *p* = .53). Across the data set, the same 5–8 top‐listed brain regions dominated the landscape, constituting >90% of the GFP^+^‐labeled cells in each mouse. These regions included ventral medial geniculate body (MGv), dorsal MG (MGd), medial MG (MGm), temporal association cortex (TeA), contralateral auditory cortex, somatosensory cortex, visual cortex, insular cortex, and BF. The proportion of input cells (calculated per mouse) from the abovementioned top‐ranked regions is plotted for the three groups in Figure 4d. Note that the dominant brain regions providing input to all three IN subtypes arise from within the auditory modality, as expected from a primary sensory region.

We detected GFP^+^ neurons in numerous other brain regions, near and far. Those regions varied in identity and numbers among mice, especially in the tail (i.e., bottom 10%) of the distribution. The full list of regions is shown in Table 1. Note that in some regions, we counted only a single‐labeled GFP^+^ neuron. While this highly skewed tail of the distribution is impossible to quantify, it nevertheless, indicates that a monosynaptic connection exists. Given the myriad brain regions and the nature of the skewed distribution (i.e., only few inputs from many regions), none of the individual regions withstood statistical power after correcting for multiple comparisons. Given this highly skewed distribution of input landscape, we first analyzed the data set in large brushstrokes. For example, we found that the fraction of local inputs versus long‐range inputs was smaller in PV neurons as compared to SST and VIP neurons (Figure 4e; *p < .05, post hoc Tukey's HSD after significant Kruskal–Wallis test). Additionally, the fraction of inputs arising from the contralateral hemisphere versus the ipsilateral hemisphere was also not significantly different among the IN types (Figure 4f; Kruskal–Wallis test, *p* = .38). Although PV neurons may be more strongly affected by long‐range inputs as compared to SST and VIP, the inputs to the three cell types were generally similar.

**TABLE 1:** Convergence indices and proportions of tracing data from individual mice

Convergence index (CI)		PV‐Cre				SST‐Cre						VIP‐Cre				Proportion		PV‐Cre				SST‐Cre						VIP‐Cre			
Regions/mouse ID		13	12	18	20	1	3	4	6	7	8	6	1	2	3	Regions/mouse ID		13	12	18	20	1	3	4	6	7	8	6	1	2	3
1	“MGv”	1.7650	8.2672	4.6988	6.0847	4.3114	3.0000	2.8602	4.7698	2.8485	4.8516	2.9279	3.5275	5.7423	4.9873	1	“MGv”	0.280	0.586	0.425	0.455	0.502	0.483	0.463	0.457	0.306	0.527	0.474	0.500	0.475	0.500
2	“AC_contra”	1.3410	1.6897	1.5807	2.8305	1.1743	0.8571	0.7634	1.3381	1.9848	1.0508	0.3243	1.0879	2.0429	1.4684	2	“AC_contra”	0.213	0.120	0.143	0.212	0.137	0.138	0.124	0.128	0.213	0.114	0.053	0.154	0.169	0.147
3	“MGd”	1.1429	1.4741	0.6894	1.6034	1.1371	0.7641	1.0000	1.6906	1.4343	1.2734	1.3604	0.8791	2.2945	1.3101	3	“MGd”	0.181	0.105	0.062	0.120	0.132	0.123	0.162	0.162	0.154	0.138	0.220	0.125	0.190	0.131
4	“TeA”	0.3963	0.4655	0.4907	0.6576	0.5057	0.3887	0.6201	0.9065	1.2475	0.6445	0.2072	0.1648	0.2086	0.1203	4	“TeA”	0.063	0.033	0.044	0.049	0.059	0.063	0.100	0.087	0.134	0.070	0.034	0.023	0.017	0.012
5	“S1”	0.1567	0.1293	1.4441	0.2441	0.0800	0.0365	0.0179	0.0360	0.0404	0.0352	0.0180	0.0714	0.2393	0.1646	5	“S1”	0.025	0.009	0.130	0.018	0.009	0.006	0.003	0.003	0.004	0.004	0.003	0.010	0.020	0.016
6	“MGm”	0.1014	0.9397	0.5901	0.3390	0.2371	0.2791	0.1505	0.3022	0.2727	0.1953	0.2342	0.3077	0.2822	0.5506	6	“MGm”	0.016	0.067	0.053	0.025	0.028	0.045	0.024	0.029	0.029	0.021	0.038	0.044	0.023	0.055
7	“V2”	0.5945	0.2241	0.1180	0.1898	0.1286	0.0365	0.0824	0.1655	0.1566	0.1797	0.0180	0.1374	0.1043	0.0696	7	“V2”	0.094	0.016	0.011	0.014	0.015	0.006	0.013	0.016	0.017	0.020	0.003	0.019	0.009	0.007
8	“InsC”	0.0000	0.1293	0.5683	0.2203	0.0886	0.0864	0.0609	0.0000	0.0000	0.0234	0.0180	0.0879	0.0920	0.4051	8	“InsC”	0.000	0.009	0.051	0.016	0.010	0.014	0.010	0.000	0.000	0.003	0.003	0.012	0.008	0.041
9	“PPC”	0.3272	0.0172	0.0590	0.1119	0.0486	0.0465	0.0072	0.0072	0.1010	0.0469	0.0541	0.1209	0.1166	0.1076	9	“PPC”	0.052	0.001	0.005	0.008	0.006	0.007	0.001	0.001	0.011	0.005	0.009	0.017	0.010	0.011
10	“BF”	0.0276	0.3534	0.1398	0.3017	0.2486	0.3223	0.2724	0.5540	0.4899	0.3789	0.5586	0.2473	0.4049	0.2278	10	“BF”	0.004	0.025	0.013	0.023	0.029	0.052	0.044	0.053	0.053	0.041	0.091	0.035	0.034	0.023
11	“S2”	0.0000	0.0431	0.2950	0.0610	0.0314	0.0365	0.0036	0.0144	0.0152	0.0078	0.0090	0.0165	0.0245	0.0823	11	“S2”	0.000	0.003	0.027	0.005	0.004	0.006	0.001	0.001	0.002	0.001	0.001	0.002	0.002	0.008
12	“SG”	0.1429	0.0086	0.0000	0.1153	0.1429	0.0565	0.0251	0.1151	0.1616	0.1328	0.1712	0.1099	0.0736	0.1519	12	“SG”	0.023	0.001	0.000	0.009	0.017	0.009	0.004	0.011	0.017	0.014	0.028	0.016	0.006	0.015
13	“Ect”	0.0323	0.1034	0.0683	0.0678	0.0686	0.1030	0.1183	0.1727	0.1414	0.1250	0.0631	0.0110	0.0061	0.0190	13	“Ect”	0.005	0.007	0.006	0.005	0.008	0.017	0.019	0.017	0.015	0.014	0.010	0.002	0.001	0.002
14	“V1”	0.0553	0.0000	0.0000	0.0407	0.0057	0.0033	0.0000	0.0000	0.0051	0.0000	0.0000	0.0165	0.0184	0.0000	14	“V1”	0.009	0.000	0.000	0.003	0.001	0.001	0.000	0.000	0.001	0.000	0.000	0.002	0.002	0.000
15	“ZI”	0.0184	0.0517	0.0248	0.0339	0.0371	0.0199	0.0287	0.0288	0.0303	0.0391	0.0360	0.0275	0.0307	0.0443	15	“ZI”	0.003	0.004	0.002	0.003	0.004	0.003	0.005	0.003	0.003	0.004	0.006	0.004	0.003	0.004
16	“TeA_contra”	0.0184	0.0345	0.0093	0.0610	0.0086	0.0033	0.0072	0.0216	0.0202	0.0195	0.0000	0.0055	0.0061	0.0000	16	“TeA_contra”	0.003	0.002	0.001	0.005	0.001	0.001	0.001	0.002	0.002	0.002	0.000	0.001	0.001	0.000
17	“VPL/VPM”	0.0323	0.0000	0.0435	0.0034	0.0057	0.0000	0.0108	0.0000	0.0000	0.0000	0.0000	0.0000	0.0000	0.0000	17	“VPL/VPM”	0.005	0.000	0.004	0.000	0.001	0.000	0.002	0.000	0.000	0.000	0.000	0.000	0.000	0.000
18	“RSA/RSG”	0.0461	0.0000	0.0031	0.0102	0.0057	0.0033	0.0036	0.0144	0.0303	0.0195	0.0000	0.0055	0.0061	0.0063	18	“RSA/RSG”	0.007	0.000	0.000	0.001	0.001	0.001	0.001	0.001	0.003	0.002	0.000	0.001	0.001	0.001
19	“S1_contra”	0.0092	0.0000	0.0559	0.0203	0.0086	0.0066	0.0036	0.0000	0.0202	0.0000	0.0000	0.0055	0.0245	0.0000	19	“S1_contra”	0.001	0.000	0.005	0.002	0.001	0.001	0.001	0.000	0.002	0.000	0.000	0.001	0.002	0.000
20	“LP”	0.0138	0.0086	0.0000	0.0475	0.0343	0.0100	0.0394	0.0000	0.0000	0.0000	0.0000	0.0495	0.0736	0.0253	20	“LP”	0.002	0.001	0.000	0.004	0.004	0.002	0.006	0.000	0.000	0.000	0.000	0.007	0.006	0.003
21	“V2_contra”	0.0230	0.0345	0.0000	0.0000	0.0371	0.0066	0.0036	0.0072	0.0000	0.0078	0.0000	0.0275	0.0123	0.0000	21	“V2_contra”	0.004	0.002	0.000	0.000	0.004	0.001	0.001	0.001	0.000	0.001	0.000	0.004	0.001	0.000
22	“ECIC”	0.0000	0.0172	0.0186	0.0305	0.0000	0.0000	0.0000	0.0000	0.0000	0.0000	0.0000	0.0055	0.0000	0.0190	22	“ECIC”	0.000	0.001	0.002	0.002	0.000	0.000	0.000	0.000	0.000	0.000	0.000	0.001	0.000	0.002
23	“M1”	0.0000	0.0086	0.0280	0.0203	0.0171	0.0066	0.0000	0.0216	0.0101	0.0000	0.0000	0.0055	0.0245	0.0063	23	“M1”	0.000	0.001	0.003	0.002	0.002	0.001	0.000	0.002	0.001	0.000	0.000	0.001	0.002	0.001
24	“MZMG”	0.0000	0.0000	0.0000	0.0610	0.0229	0.0133	0.0215	0.0000	0.0000	0.0000	0.0000	0.0275	0.0613	0.0000	24	“MZMG”	0.000	0.000	0.000	0.005	0.003	0.002	0.003	0.000	0.000	0.000	0.000	0.004	0.005	0.000
25	“OFC”	0.0000	0.0086	0.0217	0.0237	0.0000	0.0033	0.0036	0.0144	0.0101	0.0000	0.0000	0.0000	0.0061	0.0000	25	“OFC”	0.000	0.001	0.002	0.002	0.000	0.001	0.001	0.001	0.001	0.000	0.000	0.000	0.001	0.000
26	“M2”	0.0000	0.0086	0.0186	0.0271	0.0200	0.0066	0.0000	0.0072	0.0404	0.0078	0.0090	0.0055	0.0184	0.0190	26	“M2”	0.000	0.001	0.002	0.002	0.002	0.001	0.000	0.001	0.004	0.001	0.001	0.001	0.002	0.002
27	“ECIC”	0.0184	0.0000	0.0000	0.0136	0.0000	0.0000	0.0000	0.0000	0.0000	0.0000	0.0000	0.0000	0.0000	0.0000	27	“ECIC”	0.003	0.000	0.000	0.001	0.000	0.000	0.000	0.000	0.000	0.000	0.000	0.000	0.000	0.000
28	“LP_contra”	0.0138	0.0000	0.0062	0.0068	0.0029	0.0000	0.0000	0.0000	0.0101	0.0078	0.0090	0.0000	0.0245	0.0063	28	“LP_contra”	0.002	0.000	0.001	0.001	0.000	0.000	0.000	0.000	0.001	0.001	0.001	0.000	0.002	0.001
29	“PRh”	0.0000	0.0000	0.0186	0.0169	0.0114	0.0133	0.0215	0.0432	0.0253	0.0078	0.0000	0.0000	0.0061	0.0000	29	“PRh”	0.000	0.000	0.002	0.001	0.001	0.002	0.003	0.004	0.003	0.001	0.000	0.000	0.001	0.000
30	“Ect_contra”	0.0000	0.0172	0.0000	0.0102	0.0029	0.0000	0.0036	0.0000	0.0152	0.0000	0.0000	0.0000	0.0000	0.0000	30	“Ect_contra”	0.000	0.001	0.000	0.001	0.000	0.000	0.001	0.000	0.002	0.000	0.000	0.000	0.000	0.000
31	“M1_contra”	0.0000	0.0086	0.0124	0.0000	0.0086	0.0066	0.0000	0.0000	0.0000	0.0000	0.0000	0.0000	0.0000	0.0063	31	“M1_contra”	0.000	0.001	0.001	0.000	0.001	0.001	0.000	0.000	0.000	0.000	0.000	0.000	0.000	0.001
32	“VA/VL/VM”	0.0000	0.0086	0.0093	0.0034	0.0000	0.0033	0.0000	0.0000	0.0101	0.0000	0.0000	0.0055	0.0123	0.0063	32	“VA/VL/VM”	0.000	0.001	0.001	0.000	0.000	0.001	0.000	0.000	0.001	0.000	0.000	0.001	0.001	0.001
33	“AM”	0.0000	0.0000	0.0093	0.0102	0.0000	0.0000	0.0000	0.0072	0.0051	0.0000	0.0090	0.0055	0.0123	0.0063	33	“AM”	0.000	0.000	0.001	0.001	0.000	0.000	0.000	0.001	0.001	0.000	0.001	0.001	0.001	0.001
34	“Po”	0.0000	0.0000	0.0000	0.0203	0.0343	0.0100	0.0108	0.0000	0.0253	0.0078	0.0090	0.0330	0.0368	0.0759	34	“Po”	0.000	0.000	0.000	0.002	0.004	0.002	0.002	0.000	0.003	0.001	0.001	0.005	0.003	0.008
35	“MEnt”	0.0092	0.0000	0.0000	0.0000	0.0000	0.0066	0.0036	0.0000	0.0000	0.0039	0.0090	0.0000	0.0000	0.0000	35	“MEnt”	0.001	0.000	0.000	0.000	0.000	0.001	0.001	0.000	0.000	0.000	0.001	0.000	0.000	0.000
36	“InsC_contra”	0.0000	0.0000	0.0062	0.0102	0.0029	0.0000	0.0000	0.0000	0.0000	0.0000	0.0000	0.0110	0.0000	0.0000	36	“GI_contra”	0.000	0.000	0.001	0.001	0.000	0.000	0.000	0.000	0.000	0.000	0.000	0.002	0.000	0.000
37	“B”	0.0000	0.0172	0.0000	0.0000	0.0000	0.0000	0.0000	0.0000	0.0000	0.0000	0.0000	0.0000	0.0000	0.0000	37	“B”	0.000	0.001	0.000	0.000	0.000	0.000	0.000	0.000	0.000	0.000	0.000	0.000	0.000	0.000
38	“S”	0.0000	0.0086	0.0000	0.0068	0.0000	0.0000	0.0000	0.0288	0.0000	0.0000	0.0180	0.0000	0.0000	0.0000	38	“S”	0.000	0.001	0.000	0.001	0.000	0.000	0.000	0.003	0.000	0.000	0.003	0.000	0.000	0.000
39	“HDB”	0.0000	0.0000	0.0031	0.0102	0.0286	0.0133	0.0036	0.0072	0.0152	0.0195	0.0000	0.0000	0.0245	0.0063	39	“HDB”	0.000	0.000	0.000	0.001	0.003	0.002	0.001	0.001	0.002	0.002	0.000	0.000	0.002	0.001
40	“LEnt”	0.0000	0.0000	0.0031	0.0102	0.0057	0.0133	0.0036	0.0216	0.0152	0.0547	0.0000	0.0055	0.0000	0.0063	40	“LEnt”	0.000	0.000	0.000	0.001	0.001	0.002	0.001	0.002	0.002	0.006	0.000	0.001	0.000	0.001
41	“PRh_contra”	0.0000	0.0000	0.0093	0.0000	0.0057	0.0066	0.0000	0.0000	0.0101	0.0000	0.0000	0.0000	0.0000	0.0000	41	“PRh_contra”	0.000	0.000	0.001	0.000	0.001	0.001	0.000	0.000	0.001	0.000	0.000	0.000	0.000	0.000
42	“S2_contra”	0.0000	0.0000	0.0093	0.0000	0.0000	0.0000	0.0000	0.0000	0.0051	0.0000	0.0000	0.0000	0.0000	0.0000	42	“S2_contra”	0.000	0.000	0.001	0.000	0.000	0.000	0.000	0.000	0.001	0.000	0.000	0.000	0.000	0.000
43	“BMA”	0.0046	0.0000	0.0000	0.0000	0.0000	0.0000	0.0000	0.0000	0.0101	0.0000	0.0000	0.0000	0.0000	0.0000	43	“BMA”	0.001	0.000	0.000	0.000	0.000	0.000	0.000	0.000	0.001	0.000	0.000	0.000	0.000	0.000
44	“PF”	0.0046	0.0000	0.0000	0.0000	0.0000	0.0033	0.0000	0.0000	0.0000	0.0000	0.0000	0.0165	0.0245	0.0316	44	“PF”	0.001	0.000	0.000	0.000	0.000	0.001	0.000	0.000	0.000	0.000	0.000	0.002	0.002	0.003
45	“Raphe”	0.0000	0.0000	0.0062	0.0000	0.0000	0.0033	0.0000	0.0072	0.0101	0.0039	0.0180	0.0000	0.0000	0.0063	45	“Raphe”	0.000	0.000	0.001	0.000	0.000	0.001	0.000	0.001	0.001	0.000	0.003	0.000	0.000	0.001
46	“Cl”	0.0000	0.0000	0.0031	0.0034	0.0000	0.0000	0.0000	0.0000	0.0051	0.0039	0.0000	0.0000	0.0000	0.0000	46	“Cl”	0.000	0.000	0.000	0.000	0.000	0.000	0.000	0.000	0.001	0.000	0.000	0.000	0.000	0.000
47	“CA1”	0.0000	0.0000	0.0000	0.0034	0.0229	0.0033	0.0000	0.0288	0.0354	0.0117	0.0721	0.0055	0.0000	0.0000	47	“CA1”	0.000	0.000	0.000	0.000	0.003	0.001	0.000	0.003	0.004	0.001	0.012	0.001	0.000	0.000
48	“PIL”	0.0000	0.0000	0.0000	0.0034	0.0057	0.0033	0.0143	0.0072	0.0152	0.0117	0.0000	0.0000	0.0061	0.0063	48	“PIL”	0.000	0.000	0.000	0.000	0.001	0.001	0.002	0.001	0.002	0.001	0.000	0.000	0.001	0.001
49	“La”	0.0000	0.0000	0.0000	0.0000	0.0029	0.0033	0.0000	0.0432	0.0253	0.0078	0.0090	0.0000	0.0000	0.0000	49	“La”	0.000	0.000	0.000	0.000	0.000	0.001	0.000	0.004	0.003	0.001	0.001	0.000	0.000	0.000
50	“Others”	0.0046	0.0259	0.0062	0.0237	0.0429	0.0199	0.0072	0.0504	0.0101	0.0195	0.0090	0.0220	0.0245	0.0380	50	“Others”	0.001	0.002	0.001	0.002	0.005	0.003	0.001	0.005	0.001	0.002	0.001	0.003	0.002	0.004

Abbreviations: AC contra, contralateral auditory cortex; AM, anterior medial thalamic nucleus; B, basal nucleus Meynert; BF, basal forebrain; CA1, hippocampal CA1; Cl, claustrum; ECIC, external cortex of theinferior colliculus; Ect, ectorhinal cortex; HDB, nucleus of the horizontal limb of the diagonal band; InsC, insular cortex; LA, lateral amygdala; LC, locus coeruleus; LEnt, lateral entorhinal cortex; LP, lateral posterior thalamic nucleus; M1/M2, primary/secondary motor cortex; MEnt, medial entorhinal cortex; MGd, medial geniculate body dorsal part; MGm, medial geniculate body medial part; MGv, medial geniculate body ventral part; MZMG, marginal zone of the medial geniculate; OFC, orbitofrontal cortex; PF, parafascicular thalamic nucleus; PIL, posterior intralaminar thalamic nucleus; Po, posterior thalamic nucleus; PPC, posterior parietal cortex; PRh, perirhinal cortex; RSA/RSG, retrosplenial agranular cortex/ retrosplenial granular cortex; PV, parvalbumin; S, subiculum; S1/S2, primary/secondary somatosensory cortex; SG, supra geniculate body; SST, somatostatin; TeA; TeA contra, contralateral temporal association cortex; temporal association cortex; V1/V2, primary/secondary visual cortex; VA/VL/VM, ventral anterior/lateral/medial thalamic; VIP, vasoactive intestinal polypeptide.

### 2.4 Specific regional differences in long‐range connectivity onto distinct INs

Despite the abovementioned statistical challenge of the whole‐brain data, it is still possible to study specific connections if those build upon independent findings. Specifically, the function of several brain regions that connect to A1, which appeared strongly in our data, was previously studied in different contexts. For example: (1) Inputs from contralateral A1 have been suggested to induce strong PV‐mediated inhibition onto pyramidal neurons (Slater & Isaacson, 2020); (2) TeA was found to be strongly and reciprocally connected to A1, and engaged in auditory‐related plasticity (Dalmay et al., 2019; Tasaka et al., 2020); (3) auditory plasticity is strongly modulated by cholinergic inputs from BF, which exerts its function via the local inhibitory microcircuit (Froemke et al., 2007; Hangya et al., 2015; Kuchibhotla et al., 2017; Pi et al., 2013); (4) inputs from the lateral amygdala (LA) play a key role in fear conditioning via synaptic remodeling (Yang et al., 2016). Accordingly, we used these independent studies to narrow down the list of potentially interesting differences to contralateral‐A1, TeA, BF, and LA. We hypothesized that those regions will individually form distinct inputs onto different IN subtypes. To this end, we tested both the “Proportion of inputs” coming from each region with respect to the general brain‐wide landscape (Figure 4g–j), and the CI from each region separately (Figure 4k–n). While these measures are correlated, we present both since they convey slightly different meaning (see Section 3).

Inputs arising from the contralateral auditory cortex were higher for PVs but not statistically different as may have been expected from Slater and Isaacson (2020) (Figure 4g,k; [proportion] *p* = .61, Kruskal–Wallis test; [CI] *p* = .16, Kruskal–Wallis test). Further analysis, looking at the relative proportion of input neurons based on layer distribution, also revealed no compelling differences of inputs from the contralateral auditory cortex (contra AC shown in Figure S4). Inputs arising from TeA show lower connectivity onto VIP INs as compared to PV and SST (Figure 4h,l; *p < .05, post hoc Tukey's HSD after significant Kruskal–Wallis test). BF provides fewer inputs to PV neurons as compared to SST neurons (Figure 4i,m; [proportion] *p < .05, post hoc Tukey's HSD after significant Kruskal–Wallis test; [CI] *p* = .38, Kruskal–Wallis test). Finally, LA provides more inputs to SST neurons as compared to PV neurons (Figure 4j,n; *p < .05, post hoc Tukey's HSD after significant Kruskal–Wallis test). These data suggest that tracing from specific regions onto the three INs subtypes in A1 may well point to subtle differences. Functional manifestations of these differences await further experimentation.

## 3 DISCUSSION

We provide a quantitative brain‐wide list of the monosynaptic input landscape onto three major subtypes of INs in A1 of the mouse. The raw data are open for use by the community to explore and drive future hypotheses and experiments.

### 3.1 Limitations of the study

Although PV/SST/VIP are often considered three separately homogeneous IN cell groups, there is evidence that challenges this notion. Indeed, exhaustive physiological and morphological analysis of cortical neurons showed that the subdivision of cortical INs is far more fine‐grained than described by dividing INs into three (Jiang et al., 2015). Those efforts unveil as many as 15 different subcategories of INs. Our data, therefore, are inherently limited to connectivity profiles of the combined subtypes contained within each group, which are composed of more fine‐grained subtypes (Abs et al., 2018).

A second limitation of our study is inherent in the mere use of the rabies tracing technique (reviewed in (Callaway & Luo, 2015)). Rabies falls short in labeling all the inputs onto a given cell, resulting in the partial sampling of the input landscape. Our labeling resulted in an average of ∼30–40 presynaptic neurons per starter cell (PV, 31.5 ± 3.8; SST, 36.4 ± 3.6; VIP, 39.1 ± 3.7 [mean ± *SEM*]). Notably, the exact number of synapses and/or neurons impinging onto a single IN in A1 is unknown. In the hippocampus, it was argued that INs receive generally fewer inputs than excitatory neurons with significant differences among the IN subgroups (Gulyás et al., 1999). Tracing from INs in the medial prefrontal cortex showed largely similar upstream inputs to distinct INs (Sun et al., 2019). We, too, found no significant differences among the INs in their upstream input sources (Figure 3e,f). A major caveat in this estimation is that the real absolute numbers of presynaptic partners may be dramatically affected by the number of synapses each neuron makes with it. For example, if local neurons make 5–10 times more synapses with an IN as compared to long‐range inputs, this will naturally skew the numbers in the data. Thus, brain regions in Table 1 with low counts (marked as “others”) should be interpreted only as a qualitative indication of “a presence” of a long‐range connection, as we likely observe a floor effect in the tails of the distributions. Furthermore, under the assumption that rabies is not biased among the three INs, the strength of our data is in its comparative nature rather than its absolute values.

### 3.2 Similarities and differences in IN's input landscape

For a more comprehensive understanding of neural circuits, functional and anatomical studies should complement each other (Clayton et al., 2021; Malina et al., 2021; Tasaka et al., 2020). The functional properties of INs in A1 have been studied previously and indeed appear different. A partial list includes basic features like the findings that PV INs have broad receptive fields, while SST INs are narrowly tuned (Li et al., 2015; Maor et al., 2016). PV‐INs provide fast feedforward lateral inhibition, while SST‐INs inhibition is slow to develop (Li et al., 2014). VIP‐INs differ from PV‐INs in their response properties to sound intensity (Mesik et al., 2015). SSTs, but not PV‐INs, modulate surround suppression (Kato et al., 2017; Lakunina et al., 2020). The three subtypes contribute differentially to contextual information (Chen et al., 2015; Natan et al., 2015; Yarden et al., 2022). Furthermore, functional mapping of inputs suggested that thalamocortical innervation is different for PV, SST, and VIP‐INs (Ji et al., 2016). Finally, the role of PV versus SST INs is distinct in how they contribute to behavior (Masri et al., 2021). Taking all these functional studies together, we expected that the presynaptic input landscape must be different between PV, SST, and VIP. However, our results show a high degree of similarity among the subgroups in both the local and the long‐range input landscape (Figure 3). Equally surprising is the high similarity in the input landscape of excitatory and inhibitory neurons (Zhao et al., 2022), perhaps calling for a higher resolution mapping of the underlying neuronal circuitry in cortical circuits. Indeed, breaking INs into subtypes has shown that finer differences do exist (Wall et al., 2016). Another route for using whole‐brain connectivity data is to define specific hypotheses about specific circuits. Indeed, when we trimmed down the list of potential brain regions that have been suggested to connect differentially onto the inhibitory microcircuit in A1, we found differences, even when the sample is rather small (e.g., Figure 4k,l). Future studies can use such a rich anatomical landscape as a road map to study further details with a more mechanistic nature to reveal how INs contribute to cortical function.

## 4 METHODS

### 4.1 Animals

All experimental procedures were approved by the Hebrew University Animal Care and Use Committee. *PV‐Cre*, *SST‐Cre*, and *VIP‐IRES‐Cre* were obtained from the Jackson laboratories (background strain C57BL/6). We crossed with FVB mice and used an F1 hybrid of C57BL/6 and FVB strain, 8–15‐week old heterozygote female mice.

### 4.2 DNA constructs

*pAAV‐CAG‐FLEx‐oG* was constructed as described in a previous study (Vinograd et al., 2019). Briefly, *oG* was amplified by PCR from *pAAV‐EF1a‐DIO‐oG* (Addgene Plasmid #74290; RRID: Addgene_74290; a gift from Edward Callaway) (Kim et al., 2016) and then subcloned into *pAAV‐CAG‐FLEx‐RG* (Addgene Plasmid #48333; RRID: Addgene_48333) (Miyamichi et al., 2013), digested with SalI and AscI. *pAAV‐CAG‐FLEx‐ TC* ^66T^ was a gift from Liqun Luo (Addgene Plasmid #48331; RRID: Addgene_48331) (Miyamichi et al., 2013).

### 4.3 Viral procedure

AAV vectors containing *CAG‐FLEx‐TC* ^66T^ (2 × 10^12^ genomic copies per ml) and *CAG‐FLEx‐oG* (1 × 10^12^ genomic copies per ml) were produced by the ELSC vector core facility. For transsynaptic tracing, 0.1 µl of the mixture of *AAV2‐CAG‐FLEx‐TC^66T^* and *AAV2‐CAG‐FLEx‐oG* was stereotaxically injected into the left auditory cortex (coordinates relative to Bregma: anterior 2.5 mm, lateral 4.2 mm, depth 1.85 mm at 20 degrees tilt from a vertical position) by using Nanoject 2 (Drummond Scientific). EnvA‐Pseudotyped RabiesΔ*G* (5 × 10^10^ infectious particles per ml) was produced following the established protocol (Osakada & Callaway, 2013; Wickersham et al., 2007).

### 4.4 Histology

Mice were given an overdose of Pental and were perfused transcardially with phosphate‐buffered saline (PBS) followed by 4% paraformaldehyde (PFA) in PBS. Brains were postfixed for 12–24 h in 4% PFA in PBS and then cryoprotected for >24 h in 30% sucrose in PBS. Coronal slices of 50 µm were made using a freezing microtome (Leica SM 2000R) and preserved in PBS. Free floating slices were incubated for 15 min in 2.5 µg/ml of DAPI (Santa Cruz, Cat #sc‐3598) in PBS. Sections were mounted on slides and coverslipped with mounting media (Vectashield H‐1000). Sections were imaged using an Olympus IX‐81 epifluorescent microscope with a 4× and 10× objective lens (0.16 and 0.3 NA; Olympus). Images were processed in Photoshop and adjusted for contrast and brightness in each channel.

For immunostaining with anti‐PV (Figure S1b), sections were stored in PBS containing 0.01% sodium azide at 4°C. On the first day, sections were washed with 0.3% Triton‐X100 in PBS and incubated with a blocking buffer (5% gout serum, 0.3% Triton‐X100 in PBS) for 2 h. Then, the sections were incubated with primary antibody overnight in 4°C (Swant, Cat #PV 27; diluted 1:2000). On the second day, the sections were shaken with the primary antibody for 1 h in room temperature. The sections were washed with 0.3% Triton‐X100 in PBS and incubated with the secondary antibody conjugated with Alexa 488 for 2 h (Jackson ImmunoResearch; diluted 1:500). Then, the slices were washed with PBS and incubated with a DAPI solution for 15 min. Finally, the slices were washed with PBS and mounted onto slides and coverslipped with mounting media. Sections were imaged using a confocal microscope (Leica Stellaris 5) using a 20×/0.75 objective lens.

### 4.5 Rabies transsynaptic tracing

We injected AAVs (AAV2‐CAG‐FLEx‐TC^66T^ and AAV2‐CAG‐FLEx‐oG), waited 2 weeks, and then injected RVΔ*G*‐GFP into the same injection site. We sacrificed animals 5 days after rabies injection.

For the quantification of rabies tracing data, we imaged consecutively 50 µm coronal slices along with the whole brain with 4× (for long‐range input cells detection) and 10× (for starter cells and local cells detection) objectives. We counted long‐range input cells manually using a custom‐written MATLAB code. We manually registered the locations of long‐range input cells using a custom‐written MATLAB code. For counting local inputs and starter cells, we used ilastik to detect cells automatically. First, we trained ilastik with several slices to detect GFP and mCherry. Using these trained ilastik algorithms, we generated the binary mask for GFP and mCherry channels. Then we used the morphological opening function on MATLAB onto the binary masks to eliminate noise from those masks. Finally, we automatically counted the number of input cells from these masks. For detecting starter cells, we generated the binary mask, which had an overlapped fraction between GFP and mCherry channels. We counted signals that are larger than 5 pixels as starter cells. We defined cortical layers by the DAPI staining, based on the distinct density of the cells (e.g., layer 1 has few cells, layer 2 has a clear boundary, and layers 4 and 6 have brighter DAPI staining).

To construct heat maps in order to visualize the location of input/starter cells (Figures 2, 3, and S1a), we aligned slices along the *z*‐axis using the rhinal fissure and pia as reference points for the registration of geographic coordinates. In more detail, we first determined the location of the nearest pia from the input/starter cells, and the distance along the pia from that point to the rhinal fissure was set as the value of the *x*‐axis (dorsal–ventral axis). The value of the *y*‐axis was the distance from pia. We manually measured the distance from the pia for each auditory cortical layer and the layer annotation for each input/starter cell was determined by this distance of the layers. This measurement was done for each slice. Then, we superimposed all the slices using the abovementioned coordinates. To measure the distance of input cells from starter cells (Figure 3c,d), we calculated the Euclidean distance from an input cell to the centerline of pooled starter cells for each animal using the coordinates described above.

Convergence indices were calculated by the division of the number of input cells over the number of starter cells. For regional registration, boundaries were based on the Paxinos and Franklin atlas (Paxinos & Franklin, 2019).

## CONFLICTS OF INTEREST

The authors declare no conflicts of interest.

### PEER REVIEW

The peer review history for this article is available at https://publons.com/publon/10.1002/cne.25437.

## Supporting information


**Figure S1 Control experiments (related to Figures 1–4) (a)** Injection of TC66T and oG into non‐Cre animal shows no rabies‐infected cells. Micrographs are shown from one mouse. No leakage was detected in two additional mice. Scale bar, 500 µm; (b) injection of TC66T and oG into a PV‐Cre mouse, followed by staining for the PV protein. Nearly all mCherry‐positive neurons were PV‐positive. Scale bar, 100 µm

**Figure S2 Assessment of the automated cell counting (related to Figure 2). (a)** Top: Representative images and the corresponding masks calculated by the ilastik software after filtration; left: a fluorescent micrograph showing the rabies‐infected (green) and mCherry positive cells (red); center: masks; right: annotated image showing starter and input cells in red and blue, respectively. Scale bar, 100 µm; (b) no significant differences were found when comparing between the evaluated number of starter cells (left; *p* = .25, Wilcoxon signed‐rank test) and input cells (right; *p* = .25, Wilcoxon signed‐rank test) resulting from ilastik or from manual counts of the same images.

**Figure S3 Spatial analyses of input cells from PV, SST, and VIP cells in A1 along the anteroposterior axis (related to Figure 3)**. (a) Top: The fraction of the lateral distribution of input cells; bottom: heat maps of the distribution of input cells along the *A*–*P* axis; (b) the fraction of the pairwise distance of input cells from starter cells along the *A*–*P* axis. SST cells have a wider distribution of input cells as compared to PV and VIP cells (ks‐test; *p* < .0001 for all combinations).

**Figure S4 INs receive similar spatial information with regard to the layer distribution from the contralateral auditory cortex (related to Figure 4)**. The graph shows the fraction of input cells from different layers of the contralateral AC. We found no significant differences among the groups of INs.

## Data Availability

All data sets and codes used in this study are available from the corresponding author upon request or can be downloaded from https://github.com/MizrahiTeam/Tasaka‐et‐al.‐J.‐Comp.‐Neurol.‐2022/blob/main/README.md.
