# Supplementary figures and images for: The local and long‐range input landscape of inhibitory neurons in mouse auditory cortex
Source: J Comp Neurol. 2022 Dec 1;531(4):502–14. doi: 10.1002/cne.25437 (PMC10107844; doi:10.1002/cne.25437)

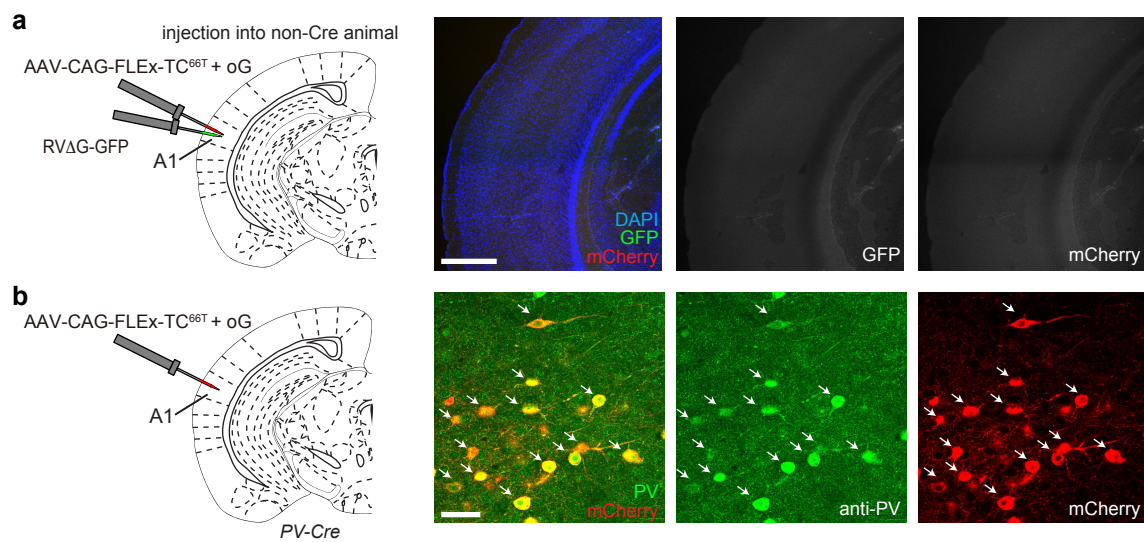

Figure S1. Tasaka et al.

Supplement: Supplementary file 1 — Figure S1 Control experiments (related to Figures 1–4) (a) Injection of TC66T and oG into non‐Cre animal shows no rabies‐infected cells. Micrographs are shown from one mouse. No leakage was detected in two additional mice. Scale bar, 500 µm; (b) injection of TC66T and oG into a PV‐Cre mouse, followed by staining for the PV protein. Nearly all mCherry‐positive neurons were PV‐positive. Scale bar, 100 µm [file CNE-531-502-s004.pdf]

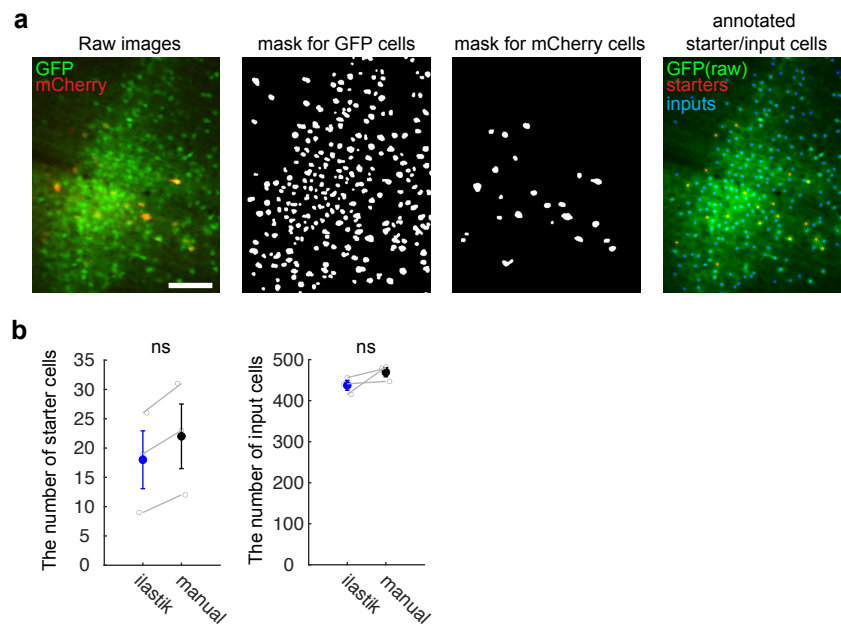

Figure S2. Tasaka et al.

Supplement: Supplementary file 2 — Figure S2 Assessment of the automated cell counting (related to Figure 2). (a) Top: Representative images and the corresponding masks calculated by the ilastik software after filtration; left: a fluorescent micrograph showing the rabies‐infected (green) and mCherry positive cells (red); center: masks; right: annotated image showing starter and input cells in red and blue, respectively. Scale bar, 100 µm; (b) no significant differences were found when comparing between the evaluated number of starter cells (left; p = .25, Wilcoxon signed‐rank test) and input cells (right; p = .25, Wilcoxon signed‐rank test) resulting from ilastik or from manual counts of the same images. [file CNE-531-502-s003.pdf]

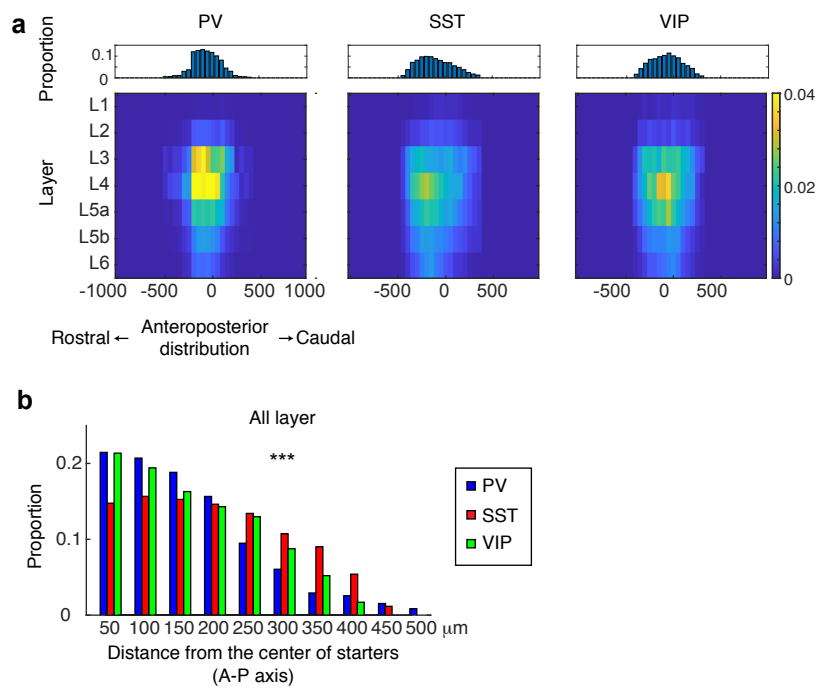

Figure S3. Tasaka et al.

Supplement: Supplementary file 3 — Figure S3 Spatial analyses of input cells from PV, SST, and VIP cells in A1 along the anteroposterior axis (related to Figure 3). (a) Top: The fraction of the lateral distribution of input cells; bottom: heat maps of the distribution of input cells along the A–P axis; (b) the fraction of the pairwise distance of input cells from starter cells along the A–P axis. SST cells have a wider distribution of input cells as compared to PV and VIP cells (ks‐test; p < .0001 for all combinations). [file CNE-531-502-s002.pdf]

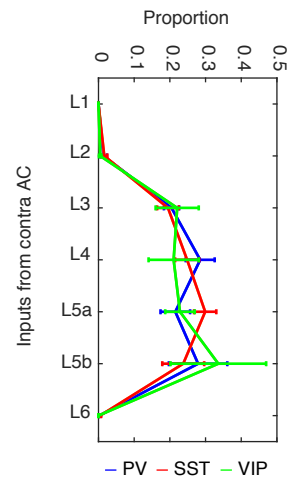

Figure S4. Tasaka et al.

Supplement: Supplementary file 4 — Figure S4 INs receive similar spatial information with regard to the layer distribution from the contralateral auditory cortex (related to Figure 4). The graph shows the fraction of input cells from different layers of the contralateral AC. We found no significant differences among the groups of INs. [file CNE-531-502-s001.pdf]
